# ICSH Recommendations for Monocyte Cell Lineage Morphologic Identification, Nomenclature Harmonization, and Utilization as a Biomarker

**DOI:** 10.1111/ijlh.70029

**Published:** 2025-11-26

**Authors:** Gina Zini, Yoon Hwan Chang, Giuseppe d'Onofrio, John Frater, Ulrich Germing, Anna Merino, Olga Pozdnyakova, David Ross, Claudio Romulo Siqueira Filho, Akiyoshi Takami, Erber Wendy

**Affiliations:** ^1^ Università Cattolica del Sacro Cuore Rome Italy; ^2^ Fondazione Policlinico Gemelli – Ematologia, Lgo Policlinico Gemelli Rome Italy; ^3^ Department of Laboratory Medicine Seoul National University Hospital Seoul Republic of Korea; ^4^ Department of Pathology and Immunology Washington University St Louis Missouri USA; ^5^ Department of Hematology, Oncology and Clinical Immunology Heinrich‐Heine‐University Düsseldorf Düsseldorf Germany; ^6^ Core Laboratory, Biochemistry and Molecular Genetics Department Hospital Clinic of Barcelona Barcelona Spain; ^7^ Department of Pathology and Laboratory Medicine Hospital of the University of Pennsylvania Philadelphia Pennsylvania USA; ^8^ Haematology Directorate SA Pathology Adelaide South Australia Australia; ^9^ Catholic University São Camilo in São Paulo São Paulo Brazil; ^10^ Division of Hematology, Department of Internal Medicine Aichi Medical University School of Medicine Nagakute Japan; ^11^ PathWest Laboratory Medicine Perth Western Australia Australia; ^12^ School of Biomedical Sciences, The University of Western Australia Perth Western Australia Australia

## Abstract

Monocytes are key components of the Mononuclear Phagocyte System, crucial in immune defense, inflammation, and tissue repair. Accurate identification and classification of monocyte lineage cells are essential for diagnosing both reactive and clonal hematologic disorders. However, morphological criteria and nomenclature inconsistencies have hindered reproducibility, particularly with the rise of automated and AI‐driven diagnostic tools. The International Council for Standardization in Haematology (ICSH) Monocyte Working Group (MWG) was convened to establish standardized morphological definitions, harmonize nomenclature for monocytes and their precursors, and evaluate their clinical utility as biomarkers. Data were collected from global laboratories, combined with an extensive literature review and consensus feedback from the ICSH General Assembly. The MWG proposes a three‐category morphological classification: blasts and blast equivalents (monoblasts and promonocytes), immature monocytes, and mature monocytes. The recommendations reaffirm the importance of morphological analysis, cytochemical staining, and flow cytometry for accurate diagnosis. Emerging automated parameters, such as monocyte distribution width (MDW), and ratios like lymphocyte/monocyte (LMR) and neutrophil/monocyte (NMR), are also recognized as valuable adjunctive biomarkers. Cytogenetic and molecular results may also impact the utilization of monocytes as a biomarker. These harmonized recommendations aim to improve diagnostic accuracy, support the development of machine learning tools, and facilitate consistent reporting across laboratories worldwide.

Monocytes are mononucleated nondividing white blood cells derived from bone marrow (BM) progenitors. They are components of the Mononuclear Phagocyte System (MPS), a term introduced by van Furth and Cohn in 1968 [1]. Since Metchnikoff's seminal description of macrophages (from Greek “big eater cells”) in 1908, our understanding has evolved to recognize now that monocytes, together with granulocytes and dendritic cells, all originate from myeloid precursors and are part of the MPS [2].

## 1 Objectives

Accurate enumeration and morphological classification of cells of the monocytic series in peripheral blood (PB) and BM represents a fundamental step for an appropriate diagnosis in reactive and clonal situations associated with an expansion of the monocytic series. Monocyte identification is currently based on morphological assessment and on cell characteristics, including cell size and cytoplasmic and nuclear characteristics. Developing and implementing machine learning and artificial intelligence (AI)‐based systems for morphological pre‐classification highlights the need for standardized definitions of these morphological criteria and harmonized nomenclature.

A Monocyte Working Group (MWG) of the International Committee for Standardization in Haematology (ICSH) was formed to improve the accuracy and reproducibility of the identification of monocyte subpopulations following evidence‐based literature. The objectives of this group of international experts were:

- To standardize the morphological criteria for monocyte and their precursors utilizing the optical microscope (OM).
- To harmonize the nomenclature for monocyte and their precursors.
- To define the diagnostic value of the quantitative and qualitative alterations of the monocytic series, integrating data from flow cytometry and molecular diagnostics.

In parallel, the MWG aims to provide a shared and harmonized basis for identification and nomenclature for automated blood cell pre‐classification systems. Based on an exhaustive analysis of the literature data on monocyte‐related parameters, we have attempted to represent the state of the art of their possible clinical utilization as predictive biomarkers, both alone and as part of AI‐driven diagnostic algorithms.

## 2 MWG Methodology

The ICSH MWG, comprising hematologists and pathologists with expertise in blood film morphology from Europe, North America, Latin America, Australia, and Asia, was established in October 2023. Each member collected anonymized data from medium to large clinical laboratories in their country regarding:

- The routine criteria for blood film review were used when there were quantitative and/or qualitative flags relating to cells of monocytic series on the blood count analyzers.
- The nomenclature used in reporting cells of the monocytic series.

This exercise demonstrated that the primary criterion used for morphological review is the presence of monocytosis, either as an isolated finding or in conjunction with other abnormalities in the blood count. Regarding the nomenclature, laboratories generally refer to published guidelines of national and international associations. These included the World Health Organization (WHO), the European LeukemiaNet (ELN), the College of American Pathologists (CAP), and the ICSH, with the WHO Classification being the most widely utilized. Despite this, non‐standardized morphologic terms were often used, including abnormal, atypical, dysplastic, reactive, and immature monocytes. These were reported to, at times, be accompanied by descriptive morphologic comments, such as regarding granular content, nuclear shape, and chromatin pattern. Based on this data and an extensive literature review, a preliminary draft of the proposed Recommendation was presented to the ICSH General Assembly in October 2024. All feedback, including comments, criticisms, and suggestions, was carefully reviewed, discussed, and evaluated by the MWG. The final recommendations, as presented in this article, reflect unanimous agreement among the members.

## 3 Monocytic Series: Biological Context

Monocytes play a vital role, particularly during infections and inflammatory responses. They are also precursors of macrophages and dendritic cells, which mainly function in phagocytosis, cytokine production, and antigen presentation. Circulating monocytes originate in the BM from a common stem cell precursor of the granulocyte‐monocyte lineage (CFU‐GM) [1, 2]. In normal physiological conditions, monoblasts and promonocytes are the mitotic compartments in the BM, taking approximately 2 days to develop from pluripotent stem cells. Monocytes are then derived from the mitotic division of promonocytes (about 1 day), representing the post‐mitotic compartment. The mature monocyte leaves the BM within about 24 h. Once released into the PB, monocytes circulate for 3–4 days (transit compartment) before migrating to the functional tissue compartment, where they persist for 1–5 weeks. A significant proportion is stored in the spleen and the lungs [3]. Monocytes respond to various stimuli, including cytokines, cellular metabolites, and microbial products, which results in their differentiation into activated tissue macrophages of the M1 or M2 type. The M1‐type macrophages support inflammatory processes, playing roles in antimicrobial defense and anti‐tumor function. The M2‐type macrophages promote T‐helper 2 (Th2)‐mediated immunity, suppress inflammatory responses, and facilitate cell repair and tissue remodeling [4]. In addition, a small population of immature mononuclear phagocytes migrates directly from the BM and continues to divide within the tissues. In healthy individuals, monocytes released from the BM into the PB are selectively restricted to nondividing cells [1, 5]. Circulating monocytes can vary significantly in size, nuclear shape, and cytoplasmic granular content [6].

The PB monocyte count increases in response to increased monocyte production in the marrow or turnover rate. It can also occur due to the mobilization of the marrow proliferative reserve compartment in response to physiological demand. This mobilization is associated with a concomitant increase in monocyte production in the marrow, resulting in the release of immature monocyte progenitors into the blood. This “left shift” is observed in patients with chronic infectious and inflammatory conditions, and it generally correlates with the monocyte count [7]. In normal circumstances, this process is controlled by cytokines and growth factors. The process can become dysregulated in pathological states, resulting in increased production or abnormal clonal expansion [8].

### 3.1 Reference Values

Monocytes have a lifespan ranging from 1 to 3 days in the blood. Monocytes usually represent approximately 1% (range: 0.3%–2.2%) of the cells detected at OM in normal BM myelograms [9, 10]; they are of Type 1 and are smaller than circulating monocytes [11]; monoblasts and promonocytes are usually rare and are difficult to identify in the BM by morphology using OM. Reference values of monocytes in the BM of healthy individuals, as assessed by flow cytometry, are higher (mean: 4.7%), likely due to both the low frequency of monocytes in the BM and the influence of PB admixture in the BM aspirate [12].

Monocytes constitute about 5% (2%–8%) of nucleated cells in the PB of healthy adults and children [13]. The normal absolute monocyte count (AMC) is reported as 0.2–0.8 × 10^9^/L in adults and 0.7–1.5 × 10^9^/L in children, without any significant ethnic variation. During the neonatal period, the mean AMC increases to 1.5 × 10^9^/L over the first 2 weeks and decreases in the third postnatal week and the subsequent months [14].

*Monocytosis* is defined as monocytes in PB more than a value that has been described between 0.8 and 1.0 × 10^9^/L [15, 16, 17]. Different types of monocytosis include:

1. *Reactive monocytosis*:
  - Transient reactive monocytosis is usually reversible. It may be secondary to viral (including COVID‐19) or parasitic infections, brucellosis, tuberculosis, listeriosis, endocarditis, granulomatous disease, autoimmune diseases, trauma, hemolytic anemia, cigarette smoking, and medications. Monocytosis may also be associated with solid tumors and nonmyeloid hematological neoplasms.
  - Persistent reactive monocytosis is seen in patients with subacute or chronic infections, storage diseases, and Rosai–Dorfman disease. It has also been reported in 0.8% of healthy older individuals and is associated with a higher frequency of clonal hematopoiesis that rarely meets diagnostic criteria for a specific neoplasm [18].
2. *Non‐reactive, possibly clonal monocytosis* (CM) refers to a persisting expansion of the monocytic mitotic compartment, leading to an increase in the AMC that persists for more than several months without an identifiable reactive cause. In the fourth edition of the WHO Classification of Hematopoietic Neoplasms (2017) [19], the diagnostic criteria were ≥ 1.0 × 10^9^/L monocytes in PB that persisted for over 3 months. This has been challenged by changes introduced by the subsequent WHO fifth classification [20, 21] and the International Consensus Classification (ICC) [22]. These have revised the diagnostic criteria for chronic myelomonocytic leukemia (CMML), allowing diagnosis if the AMC is > 0.5 × 10^9^/L, accounting for ≥ 10% of the total white blood cell (WBC) differential, and in the presence of clonality by karyotype or the presence of mutations. These cases were previously referred to as oligomonocytic CMML [23]. Juvenile chronic myelomonocytic leukemia (JCCML) may occasionally present with a monocyte count of less than 1 × 10^9^/L. [24] Clonal monocytosis can also be seen in myeloproliferative neoplasms (MPNs), in particular chronic myeloid leukemia [25] and myelodysplastic neoplasms (MDS). Clonal monocytosis of undetermined significance (CMUS) was defined in the ICC classification as persistent monocytosis (monocytes ≥ 10% of the WBC and ≥ 0.5 × 10^9^/L), associated with myeloid neoplasm‐associated mutation(s) with variant allele frequency (VAF) ≥ 2%, unexplained by a reactive condition or co‐existent hematopoietic neoplasm, without BM morphologic findings of CMML [22]. Clinical guidelines recommend that if a monocytosis persists and cannot be explained by other conditions, further investigation is required to exclude the possibility of a leukemic process [26]. Somatic mutations were detected in a study in 79% of patients with monocytosis persisting more than 2 years [27].

*Monocytopenia*, a decrease in the AMC below the normal range confirmed in at least two PB evaluations, can occur with infections, stress, autoimmune disease (e.g., systemic lupus erythematosus), aplastic anemia, genetic syndromes (e.g., GATA2 deficiency), or lymphoproliferative disease (particularly hairy cell leukemia). Also, a reduction in the monocyte count may be secondary to medications, such as immunosuppressive glucocorticoids. A reduction in circulating monocytes is typical in MDS [28]; an AMC below 0.2 × 10^9^/L highly correlates with MDS disease progression [29]. Elevated monocyte counts are also associated with a poor prognosis in low‐risk MDS [30], indicating that the AMC should be within a relatively narrow range, at least in the context of MDS.

Concerning the routine criteria for blood film review in samples with monocytosis or monocytopenia, the survey among the authors' laboratories, representing a wide range of situations worldwide, highlighted considerable heterogeneity in approaches, attributable to different local and traditional scientific attitudes. Studies aimed at standardizing these criteria have been recently published [31, 32] and represent a valuable basis for harmonization in future multi‐institutional projects. These Recommendations are therefore limited to the harmonization of morphological identification and nomenclature of cells of the monocytic lineage on blood films and BM aspirate smears.

### 3.2 Monocyte Morphology and Classification

#### 3.2.1 Historical Perspective

From Paul Ehrlich's identification of PB cells using differential staining methods in 1887 to the recognition of monocytic leukemia in 1913 (Schilling–Torgau leukemia), the morphologic identification and nomenclature of cells of the monocytic lineage have remained highly heterogeneous. This variability in morphological features stems from their intrinsic plasticity, closely linked to the complexity of their biological functions.

In 1972, Marcel Bessis proposed a harmonized approach to the morphological criteria and nomenclature for identifying monocytes [33]. He classified PB monocytes based on size, distinguishing between large (classical monocytes) and small monocytes, with diameters of 30–40 and 20–30 μm, respectively. Bessis clarified that the small monocyte had already been described but under different names, including mononuclear medium [34] referring to mononucleated cells intermediate in size between small lymphocytes and monocytes, typically observed in immune responses or specific pathological conditions; leukocytoid lymphocyte [35], histiocyte lymphocytiform [36], or flag‐like cell [37], due to the presence of pseudopods. All these cell types have been reported in conditions involving active immune responses, such as infections.

Bessis also emphasized the challenges in identifying monoblasts by OM due to their close resemblance to myeloblasts, resulting from immature chromatin and prominent nucleoli. He, therefore, suggested that accurate identification of monoblasts would require EM. In contrast, the promonocyte could be identified as having immature chromatin, nucleoli, and nuclear indentation and showing limited phagocytic activity. Bessis also linked morphologic changes with functional maturation. He noted that circulating monocytes represent an intermediate stage that subsequently fully matures into macrophages upon tissue migration [33].

In 1981, Zucker‐Franklin et al. used electron microscopy (EM) to classify mature monocytes into four subtypes based on nuclear shape and the nucleus‐to‐cytoplasm (N/C) ratio [11]. The distribution of the monocyte subtypes in the PB of healthy individuals was defined as follows:

– Type I: Round nucleus, representing < 1% of circulating monocytes. These are hardly distinguishable from lymphocytes in panoptic stains and can only be identified using cytochemical or immunohistochemical methods. Most monocytes in normal BM exhibit Type 1 morphology.
– Type II: Quadrangular nucleus, sometimes with a slight indentation on one side; they constitute 16%–18% of circulating monocytes.
– Type III: Clearly indented (horseshoe‐shaped) nucleus with coarse lobulations, maintaining a “C” shape.
– Type IV: Lobulated nucleus with two to three coarse lobulations, still preserving the C shape; represents 35%–43% of circulating monocytes.

The N/C ratio progressively decreases from Type I and II to Type III and IV, approximately from 4:1 to 2:1. The chromatin condensation increases in all four types, and nucleoli are not visible under OM. However, small micronucleoli can be identified, representing heterogeneous nuclear structures, some containing both RNA and key nucleolar proteins (nucleophosmin, nucleolin, and fibrillarin) [38].

The single‐lobed nucleus of monocytes has chromatin that becomes increasingly mature. The cells have moderate to abundant cytoplasm, often showing irregular basophilic edges and/or blunt pseudopods. Under panoptic stains, monocytes typically exhibit a chromatin pattern that can be described as delicate, lacy, diffuse, and finely stippled, distinct from that of other leukocytes. The nucleus is stained a lighter, deep bluish‐purple, while the cytoplasm is pale gray to blue with numerous reddish‐blue/lilac, very thin and lightly stained granules, giving a characteristic “ground‐glass” appearance. Cytoplasmic vacuoles may be present in type III and IV monocytes, appearing as unstained areas or “lace‐like” spaces. The nuclear contour of Types I–IV monocytes does not significantly change during reactive granulation or cytoplasmic enzyme content variations.

#### 3.2.2 Cytochemical and Ultrastructural Features

Cytochemistry maintains a crucial role in supporting the morphologic identification of the monocytic series. Nonspecific esterase (NSE) enzyme cytochemistry strongly stains the granules of both normal and leukemic monocytes. The α‐naphthyl butyrate esterase is more specific, while the α‐naphthyl acetate esterase is more sensitive. The myeloperoxidase reaction is weakly positive in monocytes and may give inconsistent staining reactions [38, 39, 40]. Both the fifth WHO classification [20, 21] and the ICC [22] of 2022 confirm the diagnostic role of cytochemistry in the differential diagnosis between clonal expansions of the monocytic series and other hematological neoplasms with confounding morphology and flow cytometry results.

Under EM, monocytes exhibit an irregular nucleus, an extensive Golgi zone, numerous ribosomes, small filaments of rough endoplasmic reticulum (RER), randomly distributed mitochondria, and occasional clusters of fibrils and small dense granules [11]. Promonocytes, in contrast, demonstrate peroxidase activity within the RER, the cisternae of the Golgi apparatus, and immature granules. Peroxidase positivity decreases as the cell matures into a monocyte, while phagocytic and microbicidal activity becomes fully functional.

#### 3.2.3 Modern Classification of Mature Monocytes: From French‐American‐British (FAB) to WHO

In 1976, the FAB classification group [41] focused on the morphology of monoblasts and promonocytes in the context of acute myeloid leukemias involving monocytic lineage expansion. The FAB group identified three major subtypes of monocytic leukemia on morphologic features: two acute and one chronic.

- Acute myelomonocytic leukemia (FAB M4), characterized by a mixture of cells with granulocytic and monocytic differentiation in varying proportions, with at least 30% blasts in PB and/or BM.
- Acute monocytic leukemia (FAB M5): Subdivided into:
  - Poorly differentiated or monoblastic leukemia (FAB M5a), dominated by large monoblasts in the BM (at least 80% of the monocytic component), sometimes also present in PB.
  - Differentiated monocytic leukemia (FAB M5b), with monoblasts (not more than 20% of the monocytic component), promonocytes and monocytes coexisting in variable proportions, with promonocytes predominating in BM and monocytes more abundant in PB than in BM.
- Chronic myelomonocytic leukemia (CMML), an MDS entity, is characterized by persistent monocytosis in PB (> 1 × 10^9^/L) and BM, with a low presence of promonocytes and monoblasts. The diagnostic workup included blood count, morphology, cytochemistry for α‐naphthyl acetate esterase (ANAE), and lysozyme estimation.

The FAB described monoblasts as large blasts, often indistinguishable from myeloblasts, characterized by a round nucleus, delicate and lacy chromatin, and one to three large, prominent vesicular nucleoli [41]. Monoblasts had abundant basophilic cytoplasm, sometimes containing rare azurophilic granules, and displayed one or more buds or pseudopodia. Promonocytes were described as having a cerebriform nucleus with a similar chromatin pattern, cytoplasm that is less basophilic with a ground‐glass appearance, and fine scattered azurophilic granules. The FAB group focused on the clonal expansion of the monocytic lineage but did not propose any criteria to distinguish clonal from reactive monocytic populations.

In 1989, Kerrigan et al. [42] described the morphology of PB monocytes in two groups of patients: those treated with recombinant granulocyte colony‐stimulating factor (rh‐GCSF) and those undergoing high‐dose chemotherapy. In rh‐GCSF‐treated patients, monocyte counts increased, resembling granulocyte kinetics with notable morphological abnormalities. Regardless of monocytosis, monocytic cells in both groups exhibited prominent vacuolation and increased N/C ratios, with immature monocytes characterized by fine chromatin and prominent nucleoli.

The first WHO classification [43] introduced the term “blast‐equivalent” for promonocytes in acute monocytic leukemia and lowered the blast percentage necessary for the diagnosis to ≥ 20%. However, incorporating promonocytes into blast counts led to increased interobserver variability, with a consequent reduction in diagnostic precision for blast enumeration under OM. In 2008, the WHO then introduced the term “abnormal monocyte” with morphological guidance to distinguish these from monoblasts and promonocytes (often difficult but not always critical) and from normal monocytes (can be difficult, but is critical) [44]. Abnormal monocytes appear “immature, yet have more condensed nuclear, convoluted or folded chromatin.”

#### 3.2.4 Morphological Standardization Efforts

In 2009, the International Working Group on Morphology of Myelodysplastic Syndrome (IWGM‐MDS) published a morphological consensus document establishing clear definitions to differentiate stages of monocyte development in both healthy individuals and pathological conditions (e.g., inflammation, infection, and leukemia) [45]. The four clinically relevant categories were: (i) Monoblasts, (ii) Promonocytes, (iii) Immature monocytes, and (iv) Mature monocytes. Notably, the group recommended the term “immature monocyte” instead of “abnormal monocyte”, aligning with the observation that chromatin immaturity can be seen in both clonal and reactive monocytic expansions.

In 2010, the ELN Morphology Consensus Faculty published a consensus glossary and statement to harmonize nomenclature and morphologic diagnostic criteria [46]. The three key subgroups of monocytic lineage cells were:

- Monoblasts and promonocytes are functionally equivalent in diagnosis; monoblasts have a round/oval nucleus, whereas promonocytes have a convoluted, folded, or grooved nucleus with a delicate chromatin pattern.
- Mature monocytes are clearly identifiable under light microscopy.
- Atypical/abnormal/reactive/immature monocytes are challenging to differentiate under OM due to high morphologic heterogeneity, which prevents a clear definition of the criteria.

Differentiating promonocytes from immature monocytes relied on the nuclear chromatin pattern and nucleoli, a reasonably reproducible distinction [47]. Cells not fitting into the monoblast/promonocyte or mature monocyte categories were to be grouped using shared terminology. The ELN proposed two main subgroups to simplify diagnostic reporting: blast percentage (monoblasts + promonocytes) and maturing monocytic series percentage (mature monocytes plus those called atypical/abnormal/reactive/immature). Furthermore, the ELN also addressed monocyte dysplasia, defining key principles:

- Dysplasia is a description, not a diagnosis;
- The term “dysplastic” applies only to nucleated myeloid lineage cells.

In 2015, the ICSH published a standardization document on PB cell nomenclature and morphology, aligning with the WHO 2008 terminology for “abnormal” and with the ELN for the use of the term “dysplastic” [48]. In 2019, the CAP issued morphological identification guidelines, notably considering monoblasts and promonocytes to be synonymous, grouping them as immature monocytes [49]. During this ICSH MWG project, the CAP Clinical Hematology and Microscopy Committee Chair formally anticipated the CAP's decision to adopt the present ICSH nomenclature at the earliest opportunity. In 2018, the Japanese Society of Laboratory Hematology (JSLH) proposed a classification system of monocytes using OM into three groups: monocytes, promonocytes, and monoblasts [50]. Differences in the proposed nomenclature for identifying diagnostic morphological subgroups within the monocytic series are summarized in Table 1.

**TABLE 1:** Differences in the proposed nomenclature for identifying diagnostic morphological subgroups of monocytic series.

	Monoblasts (blasts)	Promonocytes (blast‐equivalent)	Monocytes (mature monocytes)	Immature monocytes	Abnormal monocytes	Atypical, reactive, immature, and dysplastic
FAB 1976 [41]		YES				
WHO 2008 [44]	YES	YES				
IWGM‐MDS 2009 [45]	YES	YES	YES	YES		
ELN 2010 [46]	YES	YES	YES	YES		
ICSH 2015 [48]	YES	YES	YES		YES	
WHO 2017 [19]	YES	YES			YES	
JSLH 2018 [50]	YES	YES	YES			
CAP 2019 [49]	YES^a^	YES^a^	YES	YES^a^		
WHO 5th 2024 [20]	YES	YES		YES^b^		
ICC 2025 [22]	YES	YES		n.s^c^		
Lynch et al. 2018 [51]^d^	YES	YES	YES			YES

Abbreviations: CAP, College of American Pathologists; ELN, European LeukemiaNet; FAB, French–American–British Group; ICC, International Consensus Classification of Myeloid and Lymphoid Neoplasms; ICSH, International Council for Standardization in Hematology; IWGM‐MDS, International Working Group on Morphology of Myelodysplastic Syndrome; JSLH, Japanese Society of Laboratory Hematology; WHO, World Health Organization.

^a^ Promonocytes and Monoblasts are merged in CAP recent documents [52] into the category of Immature Monocytes, which represents a fundamental discordance from the present Recommendations (see text).

^b^ Not specified: reference to IWGM‐MDS 2009 in the chapter on Chronic Myelomonocytic Leukemia.

^c^ Not clearly specified: those cells of monocytic series that are neither blast/blast equivalent nor mature monocytes are reported as “maturing monocytes” in the chapter of Leukemia NOS.

^d^ Not harmonized nomenclature of monocytes presenting with immature‐appearing chromatin but with prominent nuclear folds or convolutions, currently reported in published papers (the cited reference is an example).

Published studies of morphological concordance [44, 45, 53, 54, 55, 56] differ significantly due to variability in group composition (experts vs. general participation), study design (multicenter vs. monocenter), diagnostic objectives (single‐cell identification vs. complete morphological diagnosis), and the lack of a shared nomenclature. The concordance values and characteristics of studies on the monocytic series in various diseases from the FAB era to the present are presented in Table 2. The mean correspondence between the reported eight studies is 72%, with variability ranging from a minimum of 50% to a maximum of 95%. The concordance between observers appears to be relatively high and tends to decrease when the group is heterogeneous.

**TABLE 2:** Concordance values and study characteristics of the monocytic series clonal diseases from the FAB era to the present.

Year	Studies	Agreement %	Participants	Support	Purpose
1993	Castoldi et al. [53]	62.6	5 expert morphologists	Glass smears (377 pts)	DGN^a^: Acute monoblastic leukemia vs. other myeloid leukemias with monoblasts
2009	IWGM‐MDS [45]	76.6	5 expert morphologists	Single digitized cells (90 cells)	Cell ID^b^: Monoblasts, promonocytes, immature monocytes, and mature monocytes
2010	ELN workup 2nd [46]	85.7	6 expert morphologists	WSI^c^: Four FOVs^d^ (66 cells)	Cell ID^b^: Monoblasts/promonocytes vs. immature/atypical monocytes
2013	ELN work up 3rd [56]	68	35 hematologists from 21 countries	WSI^c^: Four FOVs^d^ (66 cells)	Monoblasts/promonocytes vs. immature/atypical monocytes
2017	de Swart et al. [54]	50	Centers EUMDS Registry	Glass smears (4 pts)	DGN^a^: Chronic myelomonocytic leukemia
2007–2019	ELN [56]	64	280 participants^e^	Single digitized cells (288 cells)	Cell ID^b^: Monoblasts/promonocytes vs. immature/atypical monocytes
2020	Foucar et al. [55]	74.4	11 hemo‐pathologists, single center	Glass smears (20 pts)	Cell ID^b^: Abnormal monocytes (monoblasts plus promonocytes) vs. normal
2022	Zini et al. [56]	95	4 hematologists, single center	WSI (162 pts)	Cell ID^b^: Monocytic series on bone marrow

Abbreviations: ELN, European LeukemiaNet; IWGM‐MDS, International Working Group on Morphology of Myelodysplastic Syndrome.

^a^ DGN: Diagnosis.

^b^ Cell ID: Cell name identification.

^c^ WSI: Whole slide image.

^d^ FOVs: Fields of interest.

^e^ All professional profiles of doctors, biologists, and technologists from 43 countries.

#### 3.2.5 Identification of Monocyte Subpopulations Using Flow Cytometry

Since the 1980s, monoclonal antibodies have facilitated the identification of distinct monocyte subpopulations, each corresponding to different functional activities [57]. A consensus nomenclature document was established in 2010 [58]. Using CD14 and CD16 antibodies as cell markers, human monocytes were classified into three subpopulations, each associated with distinct functional roles (Figure S1):

- *MO1, Classical monocytes* (CD14^++^/CD16^−^): represent the majority of circulating monocytes (80%–90%) in healthy individuals, are typically CD14 high and CD16 negative and play a crucial role in the early immune response in phagocytosis and cytokine production (TNF‐α, IL‐1β, and IL‐6) during inflammation, migration to sites of infection or tissue damage and antigen presentation to T‐cells. A MO1 fraction above 94%, with a decrease in MO3, is a valuable marker for distinguishing CMML from reactive monocytosis and MPN‐associated monocytosis [59].
- *MO2, Intermediate monocytes* (CD14^++^/CD16^+^): represent 5%–10% of circulating monocytes and are characterized by high CD14 and low to intermediate CD16 expression. They exhibit enhanced antigen‐presenting capacity and contribute to endothelial interaction, vascular homeostasis, and sustained inflammatory responses. MO2 monocytes typically expand in sepsis (in adults), autoimmune diseases, asthma, stroke, and renal diseases [60].
- *MO3, Nonclassical monocytes* (CD14^+^/CD16^++^): represent 2%–11% of circulating monocytes and are characterized by low CD14 and high CD16 expression. They function primarily as endothelial “patrolling” cells, playing a key role in anti‐inflammation and tissue repair. Expansion is observed in disorders such as coronary artery disease and periodontitis. Nonclassical monocytes are strongly reduced in patients with hereditary diffuse leukoencephalopathy [61]. Expansion of CD16‐positive monocytes (MO2 and MO3) is observed in tuberculosis, hepatitis B and C, HIV, sepsis (children), and SARS‐CoV‐2 Infection [62].

Some authors have described morphological peculiarities in the different immunophenotype classes of monocytes. CD16^−^ monocytes (MO1) would display cytoplasmic veils, similar to the “dendritic” processes, and have a more irregular nucleus. CD16^+^ monocytes (both MO2/MO3) would exhibit an eccentric, indented nucleus with a more condensed chromatin pattern, along with small azurophilic granules suggestive of apoptosis [63]. In vitro studies reveal morphological changes during monocyte activation, with the appearance of prominent pseudopodia and amoeboid structures, particularly during differentiation into macrophage‐like cells, a process induced by ascorbic acid [64].

Despite advances in flow cytometric classification, distinctive and universally consistent morphological features for MO1, MO2, and MO3 remain undefined. Current evidence suggests that morphological characteristics are insufficient to reliably differentiate these monocyte subsets, highlighting the importance of flow cytometry for precise classification [23].

### 3.3 Automated Instrumental Parameters

#### 3.3.1 Monocyte Distribution Width (MDW)

MDW reflects the heterogeneity of monocyte volume as analyzed by different blood cell counters. MDW has recently gained attention from the scientific community. In 2018, Shen et al. reported the possibility of discriminating between patients with active pulmonary tuberculosis and healthy subjects in PB using the parameters monocyte mean volume (MMV) and MDW [65]. In the following years, studies evaluated the reliability and performance of MDW in other clinical settings, including the intensive care unit [66] and infectious diseases, such as COVID‐19‐positive patients [67] and sepsis [68]. MDW reference values (15%–21%) were evaluated on 274 blood donors using EDTA‐K2 as an anticoagulant [69].

#### 3.3.2 Cell Ratios

Lymphocyte/monocyte ratio (LMR) [70] and neutrophil/monocyte ratio (NMR) [71] have been reported as quick and inexpensive measures for predicting outcomes in patients with conditions such as infections, autoimmune diseases, cardiovascular diseases, cancers, and other infectious diseases, including COVID‐19 [72]. NMR has been observed to be significantly decreased in hematological malignancies, including CMML, compared with reactive conditions [31].

Although several studies have demonstrated the utility of instrumental monocyte measurements and ratios, further research is needed to ensure reproducibility, standardization, and inter‐instrument harmonization of these parameters [73].

Integrating AI into automated hematology analyzers has significantly improved the efficiency and accuracy of manual leukocyte differentiation in PB [74, 75]. By leveraging AI‐driven algorithms, predictive models based on monocyte‐related parameters can be developed, enhancing early disease detection and prognosis. These AI‐driven approaches have the potential to effectively support clinicians in identifying disease patterns, improving diagnostic accuracy, and streamlining personalized treatment strategies [76, 77].

### 3.4 ICSH Recommendations

The ICSH MWG, having considered this background, makes the following statements and recommendations concerning monocytes:

1. The morphological criteria for identifying monoblasts, promonocytes, and mature monocytes, as cited in the literature, have been accurately reviewed. To achieve reconciliation, simplification, and harmonization, three morphologically distinct groups within the monocytic series are proposed:
  - Blasts and blast equivalents, including monoblasts and promonocytes.
  - Immature monocytes, a heterogeneous group that does not fit the morphological criteria of blasts/blast equivalents or mature monocytes.
  - Mature monocytes.

The morphological features of these three cell types, as agreed by the ICSH MWG, are accurately described in Table 3. The term “immature” for the second type of cells in the list was selected, in agreement with the International Working Group on Morphology of Myelodysplastic Syndrome [45], from the other options (atypical and abnormal dysplastic) because it primarily describes the nuclear and cytoplasmic deviations of the cell compared to the morphology of mature monocytes and appears more neutral as for their possible causes.

2 The accurate and standardized morphological determination of monoblast and promonocyte percentages in the smear of PB and/or BM is an essential component for diagnosing and following up myeloid malignancies.
3 Blood smear morphology should be assessed per monocyte terminology (as per Recommendation no. 1 above).
4 Flow cytometry plays an important supporting role in the accurate sub‐classification of monocytes at each stage of differentiation, as well as in the detection of abnormalities and clonal populations, particularly in MDS and CMML. Genetic and molecular analyses are likely to play a significant role in this area in the immediate future.
5 NSE cytochemistry maintains a diagnostic value when morphology and flow cytometry provide uncertain results.
6 The AMC and the percentage distribution of the three monocytic subgroups in PB and BM are clinically important in diagnosis and disease classification.
7 The automated blood count parameters (MDW, LMR, and NMR) are emerging as promising, easily measurable, and cost‐effective biomarkers, particularly in cancer and inflammatory conditions. Whether used independently or integrated with other laboratory data in AI‐driven algorithms, these biomarkers can provide valuable real‐time insights into immune system components involved in inflammatory processes of various origins.

**TABLE 3:** Morphologic features and characteristics of monocytes, immature monocytes, and blasts/blast‐equivalents (monoblasts and promonocytes).

	Blasts/blasts equivalent		Immature monocytes^a^	Monocytes
	Monoblasts	Promonocytes		
Size	Large, typically 12–20 μm in diameter	Slightly smaller than monoblasts, around 10–15 μm	Heterogeneous, can be smaller or larger than monocytes	5–20 μm, one of the largest cells in PB
Nucleus	Round or oval with a fine chromatin pattern and one to two nucleoli	Indented and with a chromatin pattern analogous to monoblasts	Heterogeneous chromatin with condensed areas, irregular, convoluted. oval or rounded shape	Kidney‐ or horseshoe‐shaped, or irregular, condensed chromatin
Nucleoli	One or two	Usually absent, may be present	Usually absent	Absent
Cytoplasm	Basophilic (blue), typically agranular, though occasional fine azurophilic granules may be seen	Less basophilic than monoblasts with fine azurophilic granules	Variable color, visible granules, sometimes larger than in monocytes	Abundant, pale blue to gray, with fine azurophilic granules
Vacuoles	Occasional	May be present	Rare	Usually present
Cells that can be confused (morphology)^a^	Blasts of the other lineages, both myeloid and lymphoid	Blasts of other lineages (myeloid and lymphoid)	Reactive lymphocytes or lymphoma cells	Large and reactive lymphocytes

^a^ In all cases, the cellular context is helpful, and cytochemistry is often decisive for the difficult cell differential diagnosis.

**FIGURE 1:**
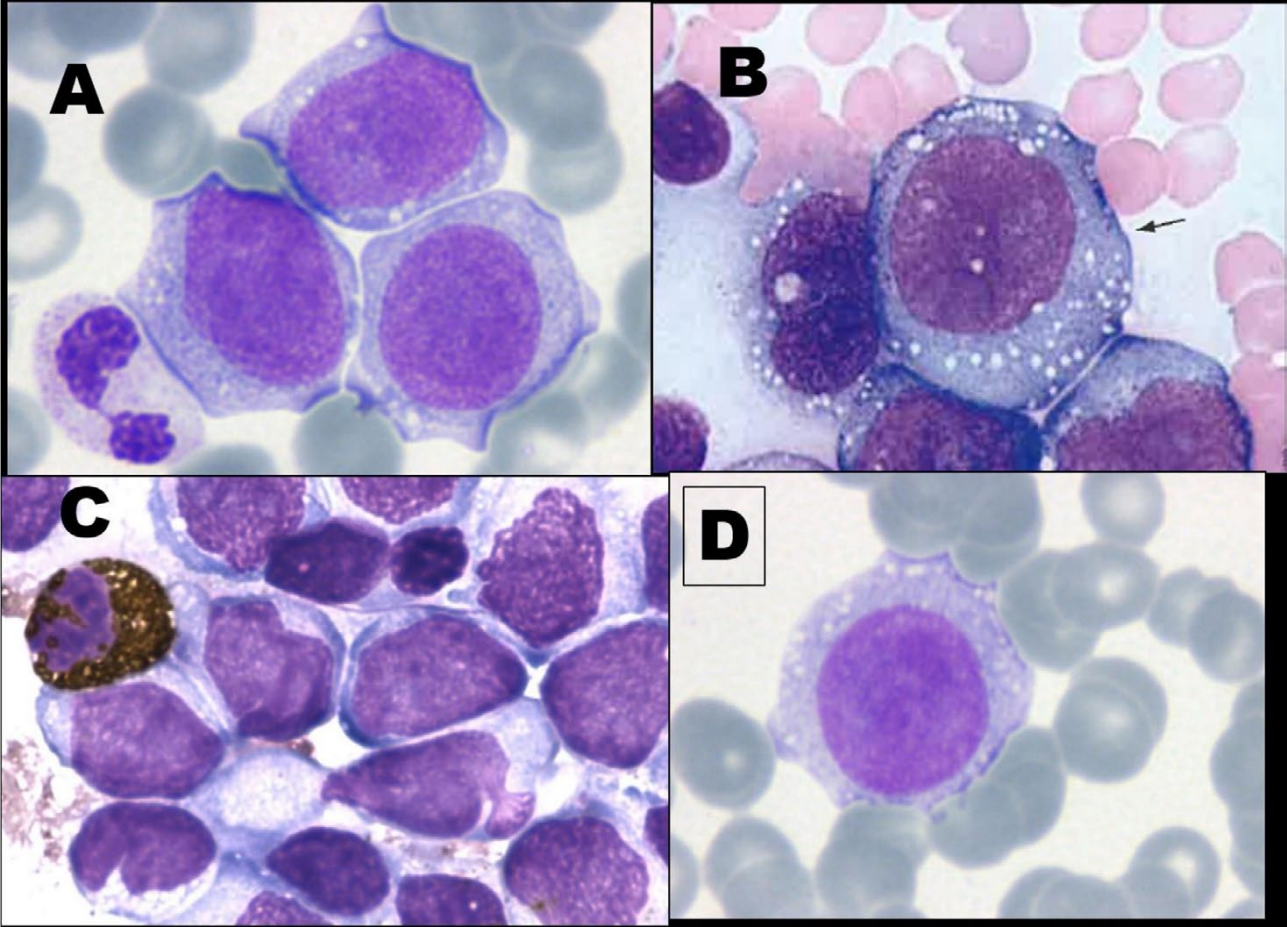
Blasts of the monocytic series. Cell images were obtained by a camera applied to conventional OM. (A, B, and D) Manual PB smearing. Stained with May–Grünwald–Giemsa (MGG) from patients with clonal neoplasms of monocytic lineage. The arrow in (B) indicates a typical monoblast. (C) Bone marrow aspirate smear, myeloperoxidase stain, showing positivity in one granulocyte on the left and negativity in all blasts. Original magnification 1000×. See Table 3 for the description of morphological details.

**FIGURE 2:**
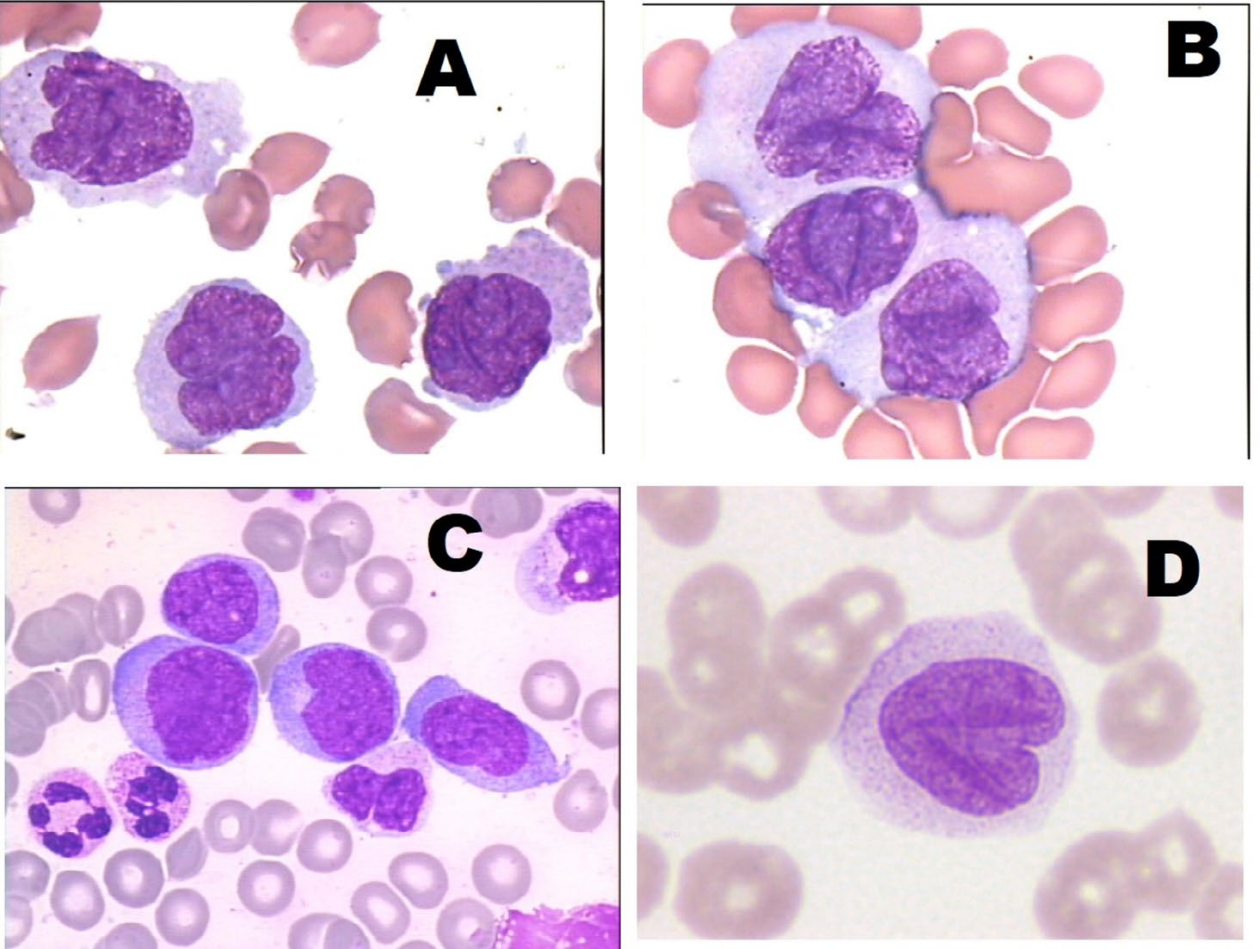
Promonocytes (blast‐equivalent) in PB smears stained by MGG at the OM. (A–D) Manual PB smearing. Stained with May–Grünwald–Giemsa (MGG) from patients with clonal neoplasms of monocytic lineage. Original magnification 1000×. See Table 3 for the description of morphological details.

**FIGURE 3:**
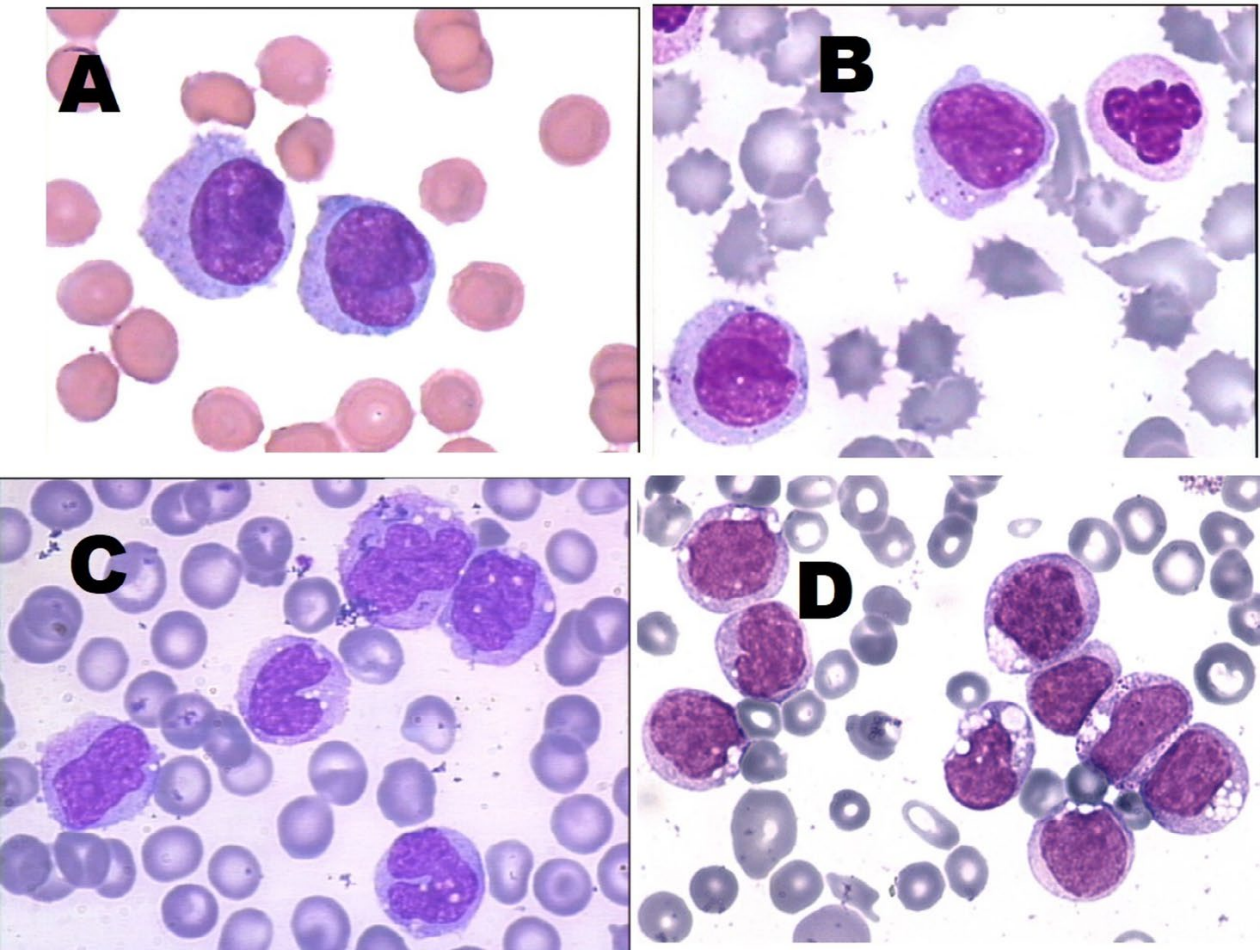
Immature monocytes from MGG‐stained PB smears at the OM from patients with inflammatory conditions (i.e., reactive) (A and B) and chronic myelomonocytic leukemia (CMML) (C and D). Original magnification 1000×. See Table 3 for the description of morphological details.

**FIGURE 4:**
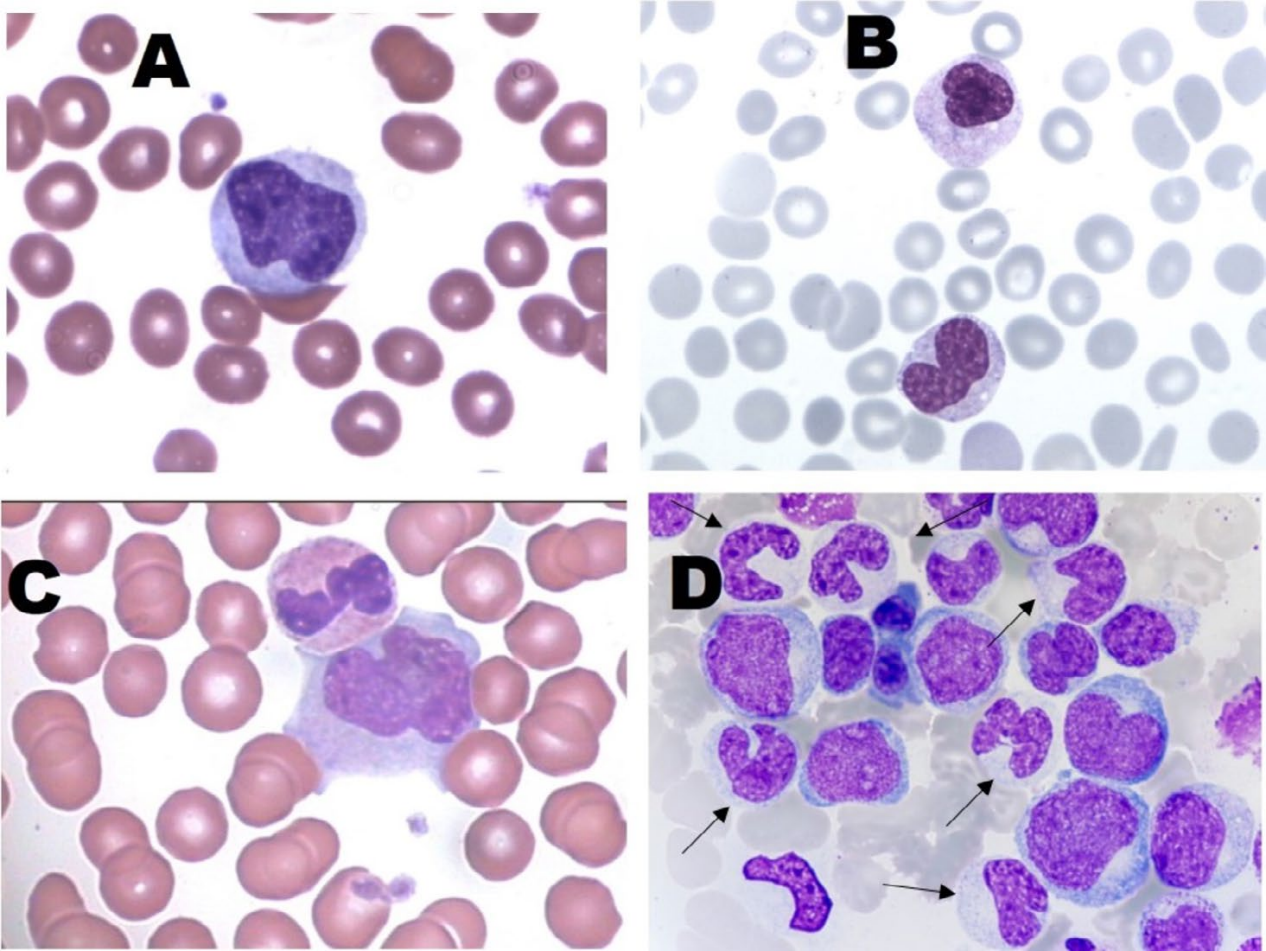
Monocytes. (A–C) Cell images obtained by a camera applied to conventional OM. Manual PB smearing stained with MGG staining. (D) Monocytes (arrows), monocyte progenitors, and two polychromatophilic erythroblasts in the BM aspirate of a patient with CMML. Original magnification 1000×. See Table 3 for the description of morphological details.

To ensure the completeness of this Recommendation, the MWG reaffirms the importance of morphological and cytochemical criteria in distinguishing different developmental stages of monocytic cells and differentiating them from morphologically similar cell lineages (Table 3). Additionally, prototype images obtained by a camera applied to conventional OM are provided as references (Figures 1, 2, 3, 4).

Finally, considering the widespread use of automatic classification systems that provide digitalized cell images, the authors have selected some digitalized cells from different systems, intending to provide a comparative tool between the classic images observed at the OM and those provided on the screen by digitalized systems (Figure S2). Examples of cytochemical stains for NSEs are visible in Figure S3.

## Author Contributions

G.Z. designed the outline and led the coordination of the manuscript. Y.H.C., G.d'O., J.F., U.G., A.M., O.P., D.R., C.R.S.F., A.T., and E.W. contributed expert content in morphological, clinical, and laboratory aspects of hematologic neoplasms. All authors participated in manuscript writing, provided critical feedback, and approved the final version for submission.

## Funding

The authors have nothing to report.

## Ethics Statement

The authors have nothing to report.

## Consent

The authors have nothing to report.

## Conflicts of Interest

Erber Wendy: Research grants from Cytek Biosciences Corporation, Cambridge University Press, Sysmex Corporation, Scopio Ltd., Thermo Fisher, AbbVie, Imago Biosciences, and patents accepted and filed in several countries. Ulrich Germing: Speakers' honorarium from AbbVie, BMS, and institutional research support from BMS, AbbVie, and Otsuka. Akiyoshi Takami: Funding from AIR WATER; lecture fees from NOVARTIS Pharmaceuticals; and donation funds from Chugai Pharmaceutical Co. Ltd., Kyowa Kirin, and Zenyaku Kogyo. Gina Zini: Lecture fees from Mindray and Siemens corporations. The other authors declare no conflicts of interest.

## Supporting information


**Figure S1:** Monocyte subsets in peripheral blood samples. Unlike the bone marrow that contains only classical monocytes, peripheral blood contains the heterogeneous monocyte populations: classical (CD14^+^CD16^−^), intermediate (CD14^+^CD16^+^), and nonclassical (CD14^−^CD16^+^). It is assumed that the intermediate and nonclassical monocytes arise once the cells have entered the circulation. Alterations of a normal state, such as infectious or neoplastic processes, alter the monocyte population ratio. (A) Normal patient. (B) Reactive monocytosis, showing an expansion of the intermediate monocyte population. (C) Chronic myelomonocytic leukemia, showing an expansion of the classical monocytes (> 94% CD14^+^CD16 classical monocytes), as they release from the bone marrow and stop the monocyte differentiation process. (D) Acute monocytic leukemia, showing the ratios similar to those of a normal patient. However, the monocyte population ratio is variable in acute monocytic leukemia.
**Figure S2:** Automated digital morphology in cells of the monocytic series. Examples of monocytic lineage PB cells are displayed on the screen from three different automated digital systems (A–E, respectively). The morphology is indicative of promonocytes (C, E in the upper left square), immature monocytes (A, D, other E squares), and monocytes (B), even though smearing and staining techniques are not standardized.
**Figure S3:** Cytochemistry for nonspecific esterases on PB smears at the OM. (A and B) Double esterase (black dots in cells of the monocytic series due to naphthyl esterase, red stain for granulocytes due to chloroacetate esterase). (C and D) Monoblasts and promonocytes display positivity for the naphthyl esterase (black).

## Data Availability

The data that support the findings of this study are available on request from the corresponding author. The data are not publicly available due to privacy or ethical restrictions.
