# Supplementary material for: ICSH Recommendations for Monocyte Cell Lineage Morphologic Identification, Nomenclature Harmonization, and Utilization as a Biomarker
Source: Int J Lab Hematol. 2025 Nov 26;48(1):12–25. doi: 10.1111/ijlh.70029 (PMC12809381; doi:10.1111/ijlh.70029)
Supplement: Supplementary file 1 — Figure S1: Monocyte subsets in peripheral blood samples. Unlike the bone marrow that contains only classical monocytes, peripheral blood contains the heterogeneous monocyte populations: classical (CD14+CD16−), intermediate (CD14+CD16+), and nonclassical (CD14−CD16+). It is assumed that the intermediate and nonclassical monocytes arise once the cells have entered the circulation. Alterations of a normal state, such as infectious or neoplastic processes, alter the monocyte population ratio. (A) Normal patient. (B) Reactive monocytosis, showing an expansion of the intermediate monocyte population. (C) Chronic myelomonocytic leukemia, showing an expansion of the classical monocytes (> 94% CD14+CD16 classical monocytes), as they release from the bone marrow and stop the monocyte differentiation process. (D) Acute monocytic leukemia, showing the ratios similar to those of a normal patient. However, the monocyte population ratio is variable in acute monocytic leukemia. Figure S2: Automated digital morphology in cells of the monocytic series. Examples of monocytic lineage PB cells are displayed on the screen from three different automated digital systems (A–E, respectively). The morphology is indicative of promonocytes (C, E in the upper left square), immature monocytes (A, D, other E squares), and monocytes (B), even though smearing and staining techniques are not standardized. Figure S3: Cytochemistry for nonspecific esterases on PB smears at the OM. (A and B) Double esterase (black dots in cells of the monocytic series due to naphthyl esterase, red stain for granulocytes due to chloroacetate esterase). (C and D) Monoblasts and promonocytes display positivity for the naphthyl esterase (black). [file IJLH-48-12-s001.docx]

**Supplemental Material**


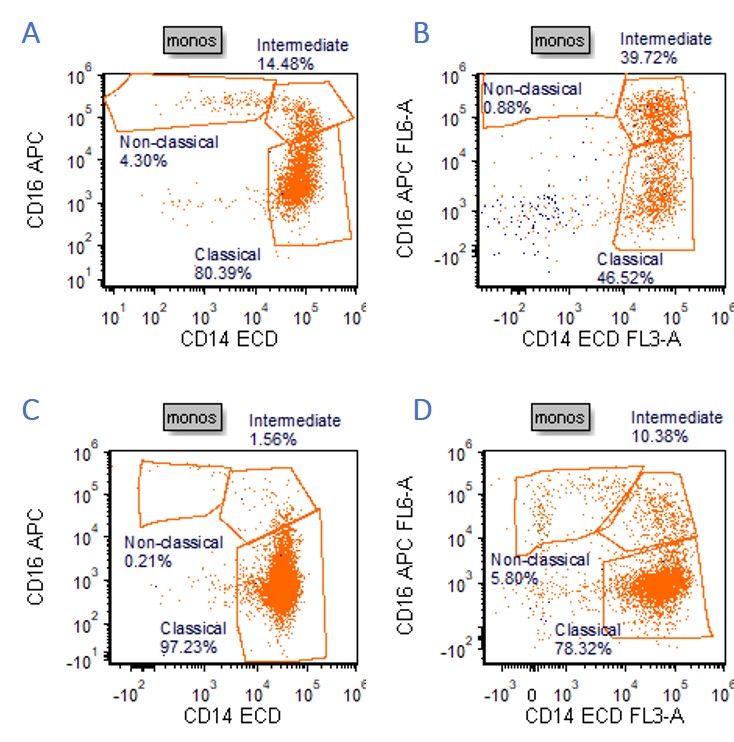


*Fig. S1 - Monocyte subsets in peripheral blood samples. Unlike the bone marrow that contains only*

*classical monocytes, peripheral blood contains the heterogeneous monocyte populations: classical*

*(CD14+ CD16-), intermediate (CD14+ CD16+) and non-classical (CD14- CD16+). It is assumed*

*that the intermediate and non-classical monocytes arise once the cells have entered the*

*circulation. Alterations of a normal state, such as infectious or neoplastic processes, alter the*

*monocyte population ratio. A. Normal patient. B. Reactive monocytosis, showing an expansion of*

*the intermediate monocyte population. C. Chronic myelomonocytic leukaemia, showing an*

*expansion of the classical monocytes (greater than 94% CD14+CD16 classical
monocytes), as they release from the bone marrow and stop the monocyte differentiation process. D. Acute monocytic leukaemia, showing the ratios similar to those of a normal patient. However, the monocyte population ratio is variable in acute monocytic leukaemia.*


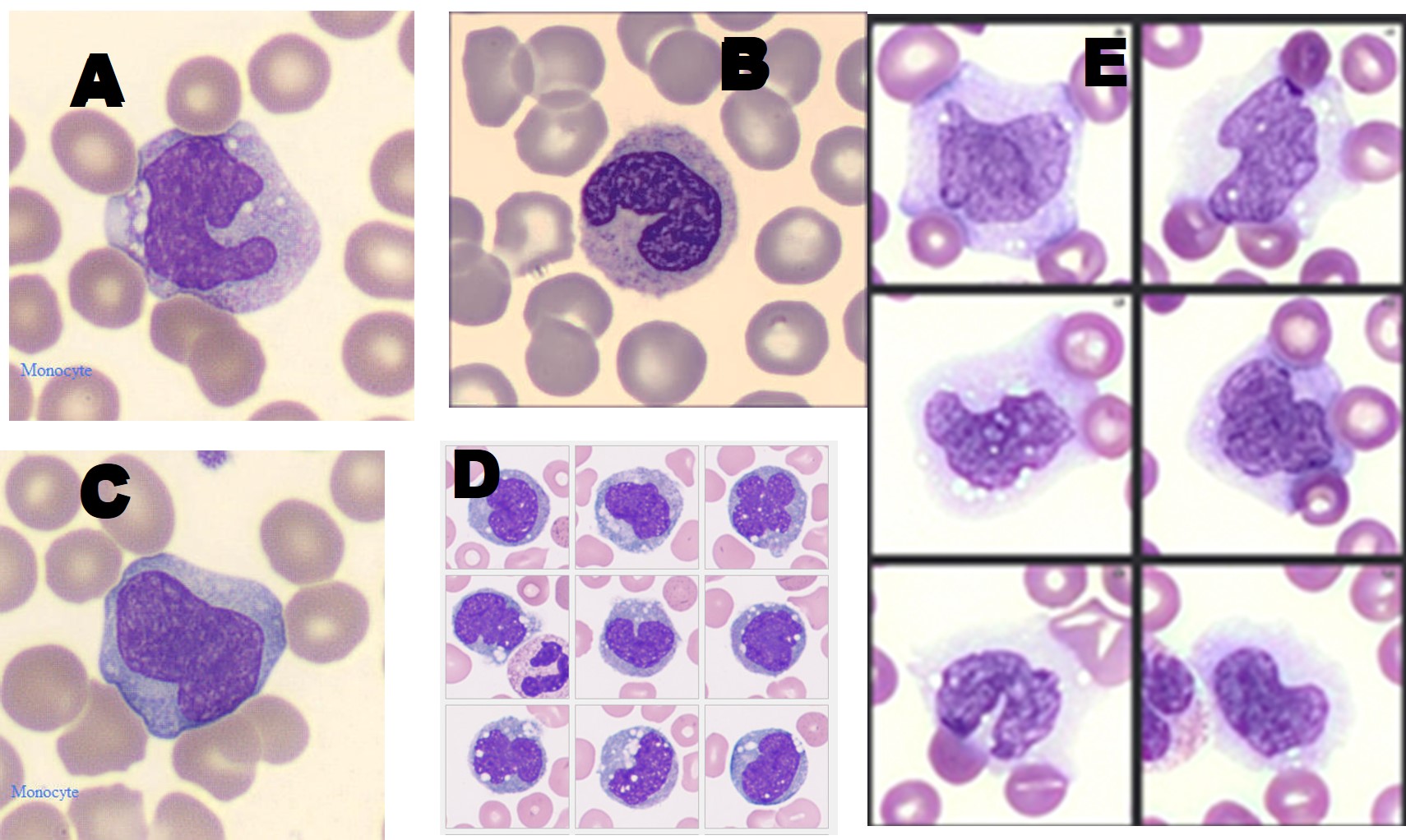


*Fig. S2 - Automated digital morphology in cells of the monocytic series. Examples of monocytic lineage PB cells are displayed on the screen from three different automated digital systems (A to C, D and E, respectively). The morphology is indicative of promonocytes (C, E in the upper left square), immature monocytes (A, D, other E squares), and monocytes (B), even though smearing and staining techniques are not standardized.*


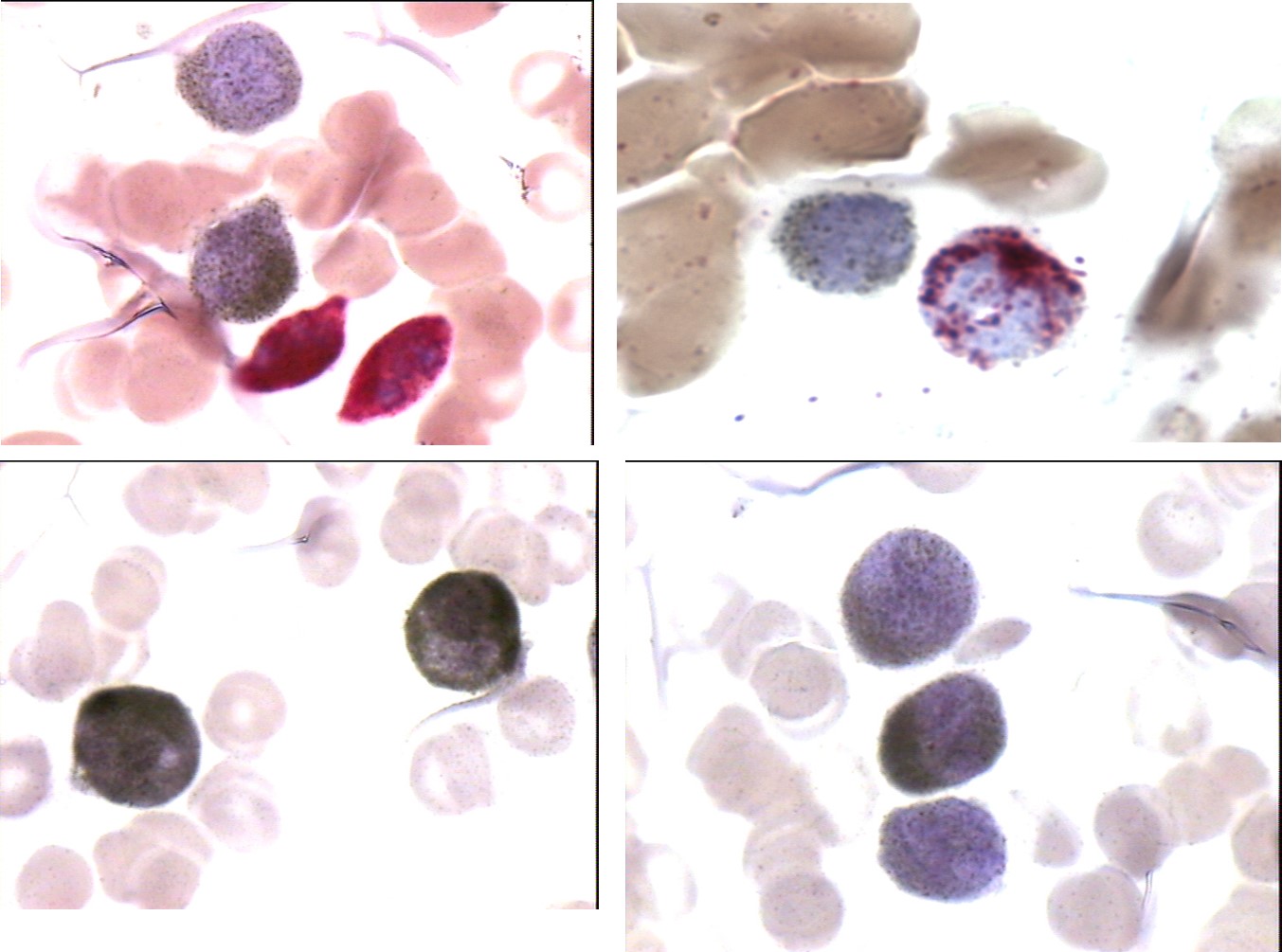


D

B

C

A

*Fig. S3 – Cytochemistry for Nonspecific esterases on PB smears at the OM. A, B Double esterase (black dots in cells of the monocytic series due to naphthyl esterase, red stain for granulocytes due to chloroacetate esterase). C, D, Monoblasts and promonocytes display positivity for the naphthyl esterase (black).*
